# Supplementary material for: Mechanochemically Synthesized PEG-OTs as a Green Corrosion Inhibitor
Source: Polymers (Basel). 2025 Feb 5;17(3):422. doi: 10.3390/polym17030422 (PMC11820882; doi:10.3390/polym17030422)
Supplement: Supplementary file 1 [file polymers-17-00422-s001.zip › polymers-3455784-supplementary.pdf]

Supplementary information

Mechanochemically Synthesized PEG-OTs as a Green Corrosion Inhibitor

Qiannian Wang†, Yuan Sang†, Jiang Yang, Hailing Liu*

Affiliation: College of Petrochemical Engineering, Liaoning Petrochemical University, Dandong Road West 1, Wanghua District, Fushun, Liaoning, 113001, China

†Qiannian Wang and Yuan Sang contribute equally in this work.

**Corresponding author email: HL2490053@outlook.com*

**Corresponding author phone number: +86 130-0926-8874*

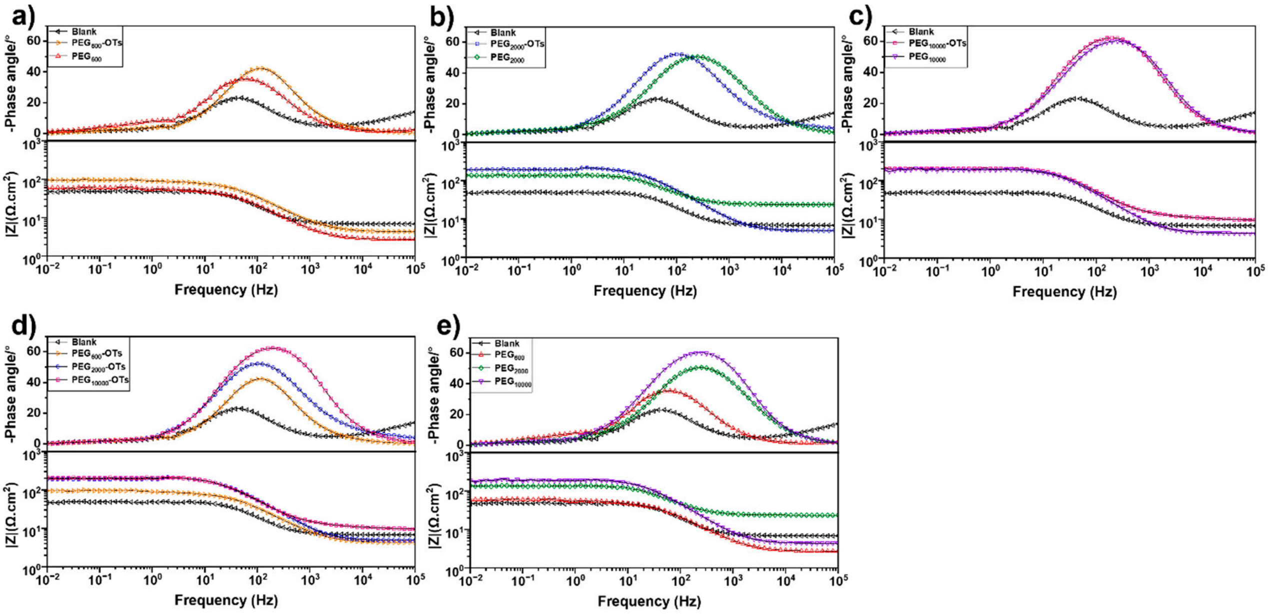

Figure S1. Bode plot of Q235 in 0.5 M HCl solution with the corrosion inhibitor of (a) PEG_{600}, PEG_{600}-OTs, (b) PEG_{2000}, PEG_{2000}-OTs, (c) PEG_{10000}, PEG_{10000}-OTs, (d) PEG_{600}, PEG_{2000}, PEG_{10000}, (e) PEG_{600}-OTs, PEG_{2000}-OTs, PEG_{10000}-OTs

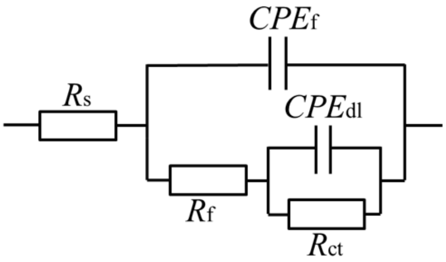

Figure S2. Equivalent Circuit Diagram: solution resistance R_s, charge transfer resistance R_{ct}, CPE_{dl}: consisting of double-layer capacitance C_d and dispersion index n_d, CPE_f: consisting of film capacitance C_f and dispersion index n_f, and film layer resistance R_f.

The EIS data are obtained by fitting a typical equivalent electric circuit model shown in Figure 6. For fitting a nonideal double-layer capacitor, the circuit components

comprise R_s the solution resistance, R_{ct} the charge transfer resistance, and CPE the constant phase element.

The CPE impedance is defined according to the following expression:

$$Z_{\mathrm{CPE}} = (Y_0^{-1}(j\omega)^{-n}) \tag{1}$$

where Z_{CPE} is CPE impedance in $\Omega{\cdot}\mathrm{cm}^{-2}$, Y_0 is CPE constant in $\mathrm{s}^n{\cdot}\Omega^{-1}{\cdot}\mathrm{cm}^{-2}$, $j = (-1)^{1/2}$ and ω is angular frequency in s^{-1}, and n is the measure of surface inhomogeneity ranging from 0 to 1. The capacitance of the double layer C_{dl} is calculated based on the charge transfer resistance as follows:

$$C_{\mathrm{dl}} = \frac{(Y_0 R_{\mathrm{ct}})^{1/n}}{R_{\mathrm{ct}}} \tag{2}$$

Then, inhibition efficiency (IE%) can be estimated from R_{ct} values as:

$$\mathrm{IE\%} = \frac{R_{\mathrm{ct1}} - R_{\mathrm{ct2}}}{R_{\mathrm{ct1}}} \times 100\% \tag{3}$$

where R_{ct1} and R_{ct2} are the charge transfer resistance in the presence and absence of the inhibitor, respectively.

The potential potentiodynamic corrosion parameters were obtained by the Tafel extrapolation method. Electrochemical parameters comprising the free potential (E_{corr}), current density (i_{corr}), polarization resistance (R_p), corrosion rate (CR), and anodic (β_a) and cathodic (β_c) slopes of Tafel branches are tabulated in Table 3. The Stern−Geary equation was followed to determine the polarization resistance (R_p):

$$\mathrm{R_P} = \frac{\beta_{\mathrm{a}}\beta_{\mathrm{c}}}{2.303 i_{\mathrm{corr}}(\beta_{\mathrm{a}} + \beta_{\mathrm{c}})} \tag{4}$$

IE% is determined from the current density (i_{corr}) as:

$$\mathrm{IE\%} = \frac{i_{\mathrm{corr1}} - i_{\mathrm{corr2}}}{i_{\mathrm{corr1}}} \times 100\% \tag{5}$$

where i_{corr1} and i_{corr2} are the corrosion current densities in the absence and presence of the inhibitor, respectively.

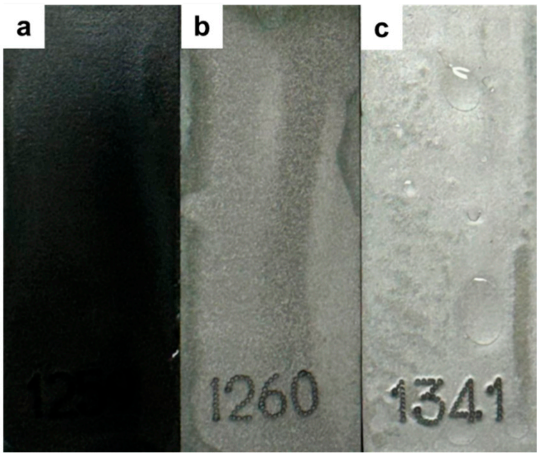

Figure S3. Q235 carbon steel pictures after immersion of 24 h in 0.5 M HCl with corrosion inhibitors of (a) blank control, (b) PEG_{10000}, and (c) PEG_{10000}-OTs

The discussion of **Figure S3** is included in the manuscript.
